# Supplementary material for: Dynamic transcriptomic profiles of zebrafish gills in response to zinc supplementation
Source: BMC Genomics. 2010 Oct 11;11:553. doi: 10.1186/1471-2164-11-553 (PMC3091702; doi:10.1186/1471-2164-11-553)
Supplement: Additional file 2 — Interactive Direct Interaction Network representing the molecular interactions between zinc, copper, iron, calcium and proteins encoded by transcripts changed by zinc supplementation. Mini web-site containing index.html and hyperlinked pages in subdirectory describing a Direct Interaction Network automatically generated based on curated interactions contained within the proprietary PathwayArchitect database. Ovals represent proteins and the circles symbolize metal ions. Objects are coloured by their abundance in zebrafish at the time-point they were significantly different from the control is a scale from -4 fold (dark green) to +4 fold (dark red). Where significant differences were found at more than one time-point, the colour overlay shows expression at the first instance. Dark blue squares denote 'binding', and light blue squares 'expression'; green squares stand for 'regulation', green diamonds for 'metabolism', and green circles for 'promoter binding'. Arrow heads indicate directionality of the interaction where annotated. All nodes and edges can be further interrogated by selecting the relative area of the image. [file 1471-2164-11-553-S2.zip › PathwayArchitect Zn xs DIN/96283.html]

# PROTEIN: STAT1

|  |  |
| --- | --- |
| Name | STAT1 |
| Type | PROTEIN |
| Description | signal transducer and activator of transcription 1, 91kDa |
| Note | The protein encoded by this gene is a member of the STAT protein family. In response to cytokines and growth factors, STAT family members are phosphorylated by the receptor associated kinases, and then form homo- or heterodimers that translocate to the cell nucleus where they act as transcription activators. This protein can be activated by various ligands including interferon-alpha, interferon-gamma, EGF, PDGF and IL6. This protein mediates the expression of a variety of genes, which is thought to be important for cell viability in response to different cell stimuli and pathogens. Two alternatively spliced transcript variants encoding distinct isoforms have been described. |
| Alias | STAT1 |
|  | signal transducer and activator of transcription-1 |
|  | ISGF-3 |
|  | Transcription factor ISGF-3 components p91/p84 |
|  | STAT91 |
|  | transcription factor ISGF-3 components p91/p84 |
|  | Stat1 |
|  | AA408197 |
|  | DKFZp686B04100 |
|  | signal transducer and activator of transcription 1 |
|  | 2010005J02Rik |
|  | Signal transducer and activator of transcription 1 |
|  | transcription factor ISGF-3 |


---

|  |  |
| --- | --- |
| GO Component | nucleus |
|  | cytoplasm |


---

|  |  |
| --- | --- |
| GO ID | GO:0007262 |
|  | GO:0003677 |
|  | GO:0007259 |
|  | GO:0009613 |
|  | GO:0006355 |
|  | GO:0005062 |
|  | GO:0005737 |
|  | GO:0005515 |
|  | GO:0007242 |
|  | GO:0004871 |
|  | GO:0003700 |
|  | GO:0007260 |
|  | GO:0005634 |
|  | GO:0007165 |
|  | GO:0000074 |
|  | GO:0006366 |
|  | GO:0007249 |
|  | GO:0006350 |
|  | GO:0005509 |
|  | GO:0006919 |
|  | GO:0009615 |


---

|  |  |
| --- | --- |
| MIM | MIM:209950 |
|  | MIM:600555 |


---

|  |  |
| --- | --- |
| Connectivity | 1608 |


---

|  |  |
| --- | --- |
| Entrez ID | 25124 |
|  | 20846 |
|  | 6772 |


---

|  |  |
| --- | --- |
| Agilent ID | A\_52\_P542540 |
|  | A\_52\_P70255 |
|  | A\_53\_P114142 |
|  | A\_14\_P111783 |
|  | A\_53\_P110552 |
|  | A\_52\_P496503 |
|  | A\_43\_P12727 |
|  | A\_23\_P56630 |
|  | A\_14\_P113301 |
|  | A\_52\_P505218 |
|  | A\_24\_P274270 |
|  | A\_14\_P137868 |
|  | A\_53\_P108036 |
|  | A\_44\_P164512 |
|  | A\_51\_P250173 |
|  | A\_52\_P70261 |


---

|  |  |
| --- | --- |
| Cellular Localization | Cytoplasm |
|  | Nucleus |
|  | Cell |
|  | Organelle |


---

|  |  |
| --- | --- |
| DbXref | KEGG pathway##04620##Toll-like receptor signaling pathway##http://www.genome.jp/dbget-bin/show\_pathway?mmu04620+20846 |
|  | KEGG pathway##04630##Jak-STAT signaling pathway##http://www.genome.jp/dbget-bin/show\_pathway?mmu04630+20846 |
|  | KEGG pathway##04630##Jak-STAT signaling pathway##http://www.genome.jp/dbget-bin/show\_pathway?rno04630+25124 |
|  | KEGG pathway##04620##Toll-like receptor signaling pathway##http://www.genome.jp/dbget-bin/show\_pathway?rno04620+25124 |
|  | KEGG pathway##04620##Toll-like receptor signaling pathway##http://www.genome.jp/dbget-bin/show\_pathway?hsa04620+6772 |
|  | KEGG pathway##04630##Jak-STAT signaling pathway##http://www.genome.jp/dbget-bin/show\_pathway?hsa04630+6772 |


---

|  |  |
| --- | --- |
| Pathway | IFN - Alpha Signalling |
|  | PDGF Signaling |
|  | MAPK Signaling |
|  | P38 Signaling |
|  | JAK-STAT Signaling |
|  | EGF Signaling |
|  | TGF-beta |
|  | Interferon-alpha Signaling |
|  | Zn xs inventory |
|  | Zn xs DIN |


---

|  |  |
| --- | --- |
| GO Process | response to virus |
|  | STAT protein nuclear translocation |
|  | regulation of transcription, DNA-dependent |
|  | tyrosine phosphorylation of STAT protein |
|  | transcription |
|  | I-kappaB kinase/NF-kappaB cascade |
|  | caspase activation |
|  | transcription from RNA polymerase II promoter |
|  | JAK-STAT cascade |
|  | signal transduction |
|  | intracellular signaling cascade |
|  | response to pest, pathogen or parasite |
|  | regulation of progression through cell cycle |


---

|  |  |
| --- | --- |
| UniGene | Mm.277406 |
|  | Hs.470943 |
|  | Rn.33229 |


---

|  |  |
| --- | --- |
| Affymetrix Probeset ID | 101465\_at |
|  | 114635\_at |
|  | 1368835\_at |
|  | 1372757\_at |
|  | 1387354\_at |
|  | 1420915\_at |
|  | 1450033\_a\_at |
|  | 1450034\_at |
|  | 200887\_s\_at |
|  | 209969\_s\_at |
|  | 232375\_at |
|  | 32859\_at |
|  | 32860\_g\_at |
|  | 33338\_at |
|  | 33339\_g\_at |
|  | 61191\_at |
|  | AFFX-HUMISGF3A/M97935\_3\_at |
|  | AFFX-HUMISGF3A/M97935\_5\_at |
|  | AFFX-HUMISGF3A/M97935\_MA\_at |
|  | AFFX-HUMISGF3A/M97935\_MB\_at |
|  | g12803734\_3p\_a\_at |
|  | g6274551\_3p\_s\_at |
|  | Hs.137447.0.S1\_3p\_at |
|  | M97935\_s\_at |
|  | M97936\_at |
|  | Msa.289.0\_s\_at |
|  | rc\_AA892553\_at |
|  | rc\_AI105268\_at |
|  | 1440481\_at |
|  | TC16691\_at |
|  | TC31356\_at |
|  | TC34761\_at |
|  | TC34761\_g\_at |
|  | TC35172\_s\_at |
|  | rc\_AA849827\_g\_at |
|  | rc\_AA999171\_at |


---

|  |  |
| --- | --- |
| GO Function | protein binding |
|  | transcription factor activity |
|  | hematopoietin/interferon-class (D200-domain) cytokine receptor signal transducer activity |
|  | DNA binding |
|  | calcium ion binding |
|  | signal transducer activity |


---

|  |  |
| --- | --- |
| Nucleotide | BC002704 |
|  | BC057690 |
|  | BC004808 |
|  | U18664 |
|  | AC067945 |
|  | CR749636 |
|  | NM\_139266 |
|  | AK088874 |
|  | AK039458 |
|  | AK041599 |
|  | M97936 |
|  | NM\_032612 |
|  | U18666 |
|  | AK157458 |
|  | AK157263 |
|  | AY366464 |
|  | U18665 |
|  | U18662 |
|  | AK018544 |
|  | NM\_009283 |
|  | AF349678 |
|  | U80267 |
|  | AA960121 |
|  | U18667 |
|  | U18668 |
|  | AK096686 |
|  | U06924 |
|  | AF053767 |
|  | AK041814 |
|  | U18670 |
|  | BG699050 |
|  | AK159887 |
|  | AY865620 |
|  | BT007241 |
|  | M97935 |
|  | BC042551 |
|  | BC062079 |
|  | U18669 |
|  | U18663 |
|  | AK008127 |
|  | AK082706 |
|  | AF205604 |
|  | AK046517 |
|  | AF182311 |
|  | NM\_007315 |
|  | AK084855 |


---

|  |  |
| --- | --- |
| Protein | AAW56072 |
|  | CAH18430 |
|  | BAE35456 |
|  | BAC38579 |
|  | AAH57690 |
|  | BAC30355 |
|  | AAH42551 |
|  | NP\_009330 |
|  | NP\_033309 |
|  | AAK48844 |
|  | P42225 |
|  | BAE43313 |
|  | BAC39293 |
|  | AAA19454 |
|  | AAH04808 |
|  | BAB31265 |
|  | AAR13284 |
|  | AAB64012 |
|  | AAP35905 |
|  | BAE43314 |
|  | NP\_116001 |
|  | AAH02704 |
|  | BAE34017 |
|  | P42224 |
|  | BAC32766 |
|  | NP\_644671 |
|  | AAY24183 |
|  | AAF20200 |
|  | AAC08438 |
|  | AAH62079 |


---

|  |  |
| --- | --- |
| Organism | Mammal |


---

|  |  |
| --- | --- |
| Location | chromosome 1, 1 25.9 cM, 1 C1.1 (Mus musculus) |
|  | chromosome 2, 2q32.2 (Homo sapiens) |
|  | chromosome 9, 9q31-q32 (Rattus norvegicus) |
|  | 1 25.9 cM (Mus musculus) |


---

|  |  |
| --- | --- |
